# Application of laboratory micro X-ray fluorescence devices for X-ray topography

**DOI:** 10.1107/S1600576724003509

**Published:** 2024-05-17

**Authors:** Christo Guguschev, Christian Hirschle, Kaspars Dadzis, Albert Kwasniewski, Michael Schulze, Leonard Schellkopf, Carsten Richter

**Affiliations:** a Leibniz-Institut für Kristallzüchtung, Max-Born-Strasse 2, 12489 Berlin, Germany; b Bruker Nano GmbH, Am Studio 2D, 12489 Berlin, Germany; Oak Ridge National Laboratory, USA; North Carolina State University, USA

**Keywords:** X-ray topography, single crystals, energy-dispersive Laue mapping, Lang topography, Si crystal growth

## Abstract

It is demonstrated that high-resolution energy-dispersive X-ray fluorescence mapping devices based on a micro-focused beam are not restricted to high-speed analyses of element distributions or to the detection of different grains, twins and subgrains in crystalline materials but can also be used for the detection of dislocations in high-quality single crystals. Si single crystals with low dislocation densities were selected as model materials to visualize the position of dis­locations by the spatially resolved measurement of Bragg-peak intensity fluctuations. These originate from the most distorted planes caused by the stress fields of dislocations. The results obtained by this approach are compared with laboratory-based Lang X-ray topographs. The presented methodology yields comparable results and it is of particular interest in the field of crystal growth, where fast chemical and microstructural characterization feedback loops are indispensable for short and efficient development times. The beam divergence was reduced via an aperture management system to facilitate the visualization of dislocations for virtually as-grown, non-polished and non-planar samples with a very pronounced surface profile.

## Abstract 2

A laboratory X-ray topography technique is presented. One of the advantages of this technique is that it can be used to visualize dislocations in non-polished and non-planar crystals with a very pronounced surface profile.

## 1. Introduction

X-ray topography (XRT) is a powerful technique for imaging defects in single-crystalline materials that are pivotal for science and technology (Suvorov, 2018 ▸; Danilewsky, 2020 ▸). Typically, collimated X-rays (characteristic or white radiation) impinging on a single-crystalline sample are diffracted, if the Bragg condition is satisfied, at a specific angle onto a high-resolution X-ray film or a two-dimensional (*e.g.* CCD) detector. An image of the crystal is obtained, given that the latter exhibits only small lattice inhomogeneities, such that the diffracted X-rays leaving the crystal are nearly parallel and hence lead to a one-to-one correspondence of sample position and detector pixel. Here, the tolerance of the angular variation is defined by the solid angle of the detector pixels and is therefore increased by a short sample-to-detector distance.

The imaging of defects in crystals becomes possible due to the distortion of lattice planes by their stress fields. The X-ray diffraction process is very sensitive to these distortions, leading to additional contrast even if the defects are several orders of magnitude smaller than the spatial resolution of XRT. On this basis, a variety of experimental XRT techniques have been developed, as summarized *e.g.* by Lider (2021 ▸). Recent developments show that a high-resolution 3D view of the defect distribution can be obtained using a focused sheet-shaped beam (Yoneyama *et al.*, 2023 ▸; Yildirim *et al.*, 2023 ▸) or a rotation about the lattice-plane normal (Hänschke *et al.*, 2017 ▸; Straubinger *et al.*, 2023 ▸). Using monochromatic X-rays further­more allows a high level of quantification of lattice inhomogeneities and characteristic strain fields caused by these defects (Guguschev *et al.*, 2022 ▸; Tran Caliste *et al.*, 2023 ▸).

In addition, using the full-field character of 2D XRT imaging facilitates time-resolved *in situ* measurements that allow the study of plasticity or crystallization (Dresselhaus-Marais *et al.*, 2021 ▸; Becker *et al.*, 2019 ▸). Although the transmission of X-rays through the crystal under study raises an important issue for the optimization of XRT measurements, the technique is universally applied to a wide variety of materials like silicon (Stockmeier *et al.*, 2017 ▸), silicon carbide (Fujie *et al.*, 2021 ▸), group III nitrides (Kirste *et al.*, 2021 ▸; Hartmann *et al.*, 2023 ▸), gallium oxide (Yao *et al.*, 2023 ▸) and diamond (Shikata *et al.*, 2021 ▸).

In contrast to the full-field imaging techniques discussed above, scanning of a focused beam across the sample while picking up the diffraction signal is an alternative approach to obtaining XRT data. If a polychromatic beam is used, several Bragg reflections can be detected for each illuminated spot, giving information about the strain state of the crystallographic unit cell. Such an approach is used in the field of Laue micro-diffraction (µ-Laue) (Robach *et al.*, 2011 ▸; Puru­shot­tam Raj Purohit *et al.*, 2022 ▸). As an alternative to position-sensitive detectors, energy-dispersive zero-dimensional (‘point’) detectors are used to map lattice inhomogeneities and strain at synchrotron radiation sources [see *e.g.* Simpson *et al.* (2019 ▸)]. A focused X-ray beam and ‘point’ detectors are nowadays also implemented in low-cost compact laboratory devices for X-ray fluorescence (XRF) mapping. However, to the best of our knowledge, use of these laboratory devices for defect imaging in single crystals is not yet established.

In this paper, we show that laboratory-based scanning techniques using polychromatic radiation, focused to spot sizes in the range of 5–25 µm, are capable of visualizing individual defects such as dislocations. This is an extension of the energy-dispersive Laue mapping (EDLM) technique (Guguschev *et al.*, 2015 ▸), which has proven capabilities to detect low- and high-angle grain boundaries, twins and striations in crystalline materials (Guguschev *et al.*, 2019 ▸; Buegler *et al.*, 2021 ▸; Subramanian *et al.*, 2023 ▸).

## 2. Experimental

**Figure 1:**
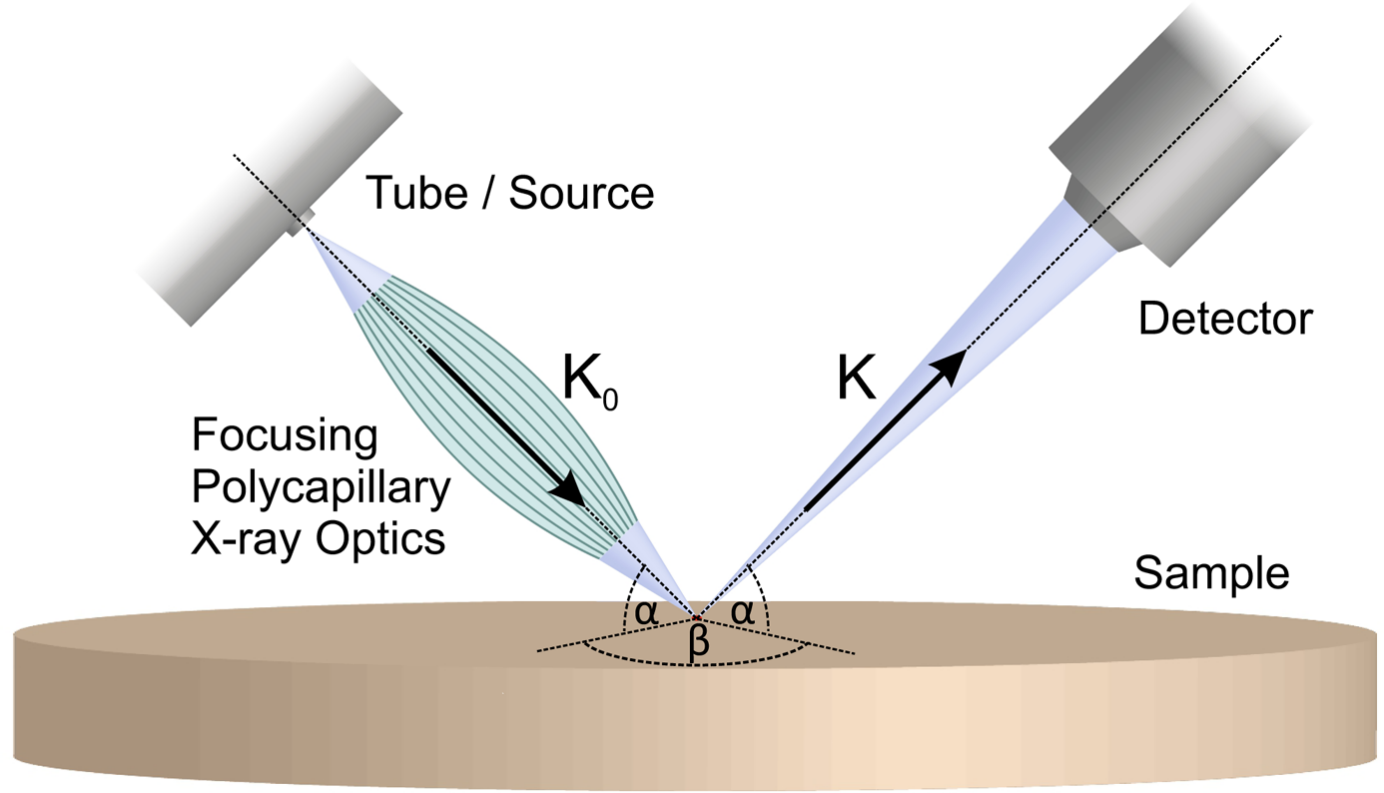
A schematic diagram of the µ-XRF setup for a planar sample (Guguschev *et al.*, 2015 ▸).

The investigations were performed at Leibniz-Institut für Kristallzüchtung (IKZ) using a µ-XRF spectrometer prototype M4 TORNADO PLUS with an aperture management system (AMS) provided by Bruker Nano GmbH, Berlin, Germany. The measurement system was equipped with a Rh X-ray source operated at 50 kV and 600 µA. The primary radiation was focused on the sample surface by a polycapillary X-ray lens at an angle of 50° (α in Fig. 1 ▸).

The minor axis of the elliptical measurement spot was about 17 µm at 17.4 keV (Mo *K*α) and 31 µm at 2.3 keV (Mo *L*α). This was determined from a mapping over the edge of a molybdenum foil, where the spot size was defined as the measured distance between 80 and 20% of the intensity of characteristic X-rays from Mo. The X-ray spectrum of fluorescence and diffracted peaks was detected using a circular silicon drift detector (SDD) with high energy resolution [<145 eV at 5.893 keV (Mn *K*α)] and a detection area of 30 mm^2^. The detector was also tilted by 50° and rotated by 90° around the normal of the surface (angle β in Fig. 1 ▸).

**Figure 2:**
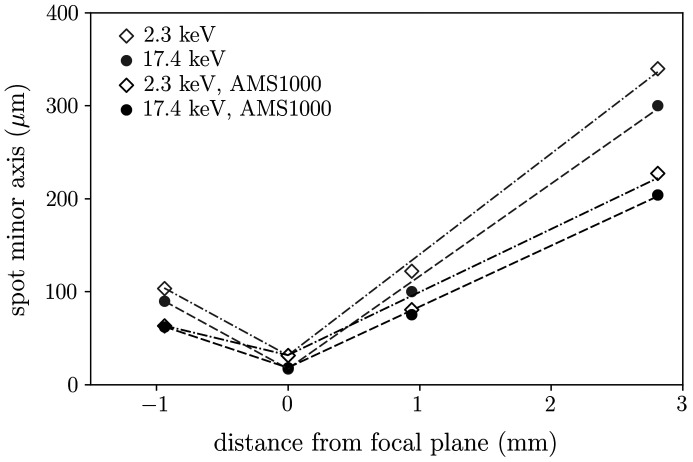
Minor axis of the spot as a function of the vertical distance between the horizontal focal plane of the beam and the measurement plane. Positive distances indicate increasing distance to the polycapillary lens. AMS1000 indicates that a 1000 µm aperture was used.

In an additional EDLM mapping, the AMS was used to place an aperture of 1000 µm diameter between the tube and the lens to reduce the divergence of the beam. Without the AMS, the minor axis of the measurement spot at 17.4 keV increases on average by 100 µm mm^−1^ for the first 3 mm of movement away from the horizontal focal plane (see Fig. 2 ▸). With the 1000 µm aperture in place, this decreases to 66 µm mm^−1^, while the intensity of the measured spectrum is also decreased.

The µ-XRF system was used to map the surface of Si crystals (see Table 1 ▸ for measurement conditions). For each spot on the surface, the measurement yields a spectrum of emitted X-rays in the range 0.2–40 keV. A longitudinal section chemo-mechanically polished on both sides (sample Si-1) was prepared from a Dash neck grown from the melt using the silicon granulate crucible (Si-GC) technique (Dadzis *et al.*, 2020 ▸). The thickness of the sample was about 500 µm and Lang topography images were available for a direct comparison. The Lang method (Lang, 1959 ▸) in transmission geometry is one of the most widely used techniques to visualize dis­locations and other defects.

The residual part of the Dash neck (sample Si-3) next to the polished sample was subjected to an acid mixture of HF (40%) and HNO_3_ (69%) in a ratio of 1:3 at room temperature for 2 min to remove about 200 µm of the periphery of the sample. As a final step, the sample was rinsed with ultrapure water. The main part of this sample (where most of the dislocations are expected) was measured with the cut surface placed on the stage and an aperture of 1000 µm to compensate for the pronounced surface profile by lowering the beam divergence (Fig. 2 ▸).

A longitudinal section of crystal, chemo-mechanically polished on both sides (sample Si-2) with a thickness of 2 mm, was prepared from the shoulder part of a float-zone grown Si crystal, where the growth conditions were not optimized, leading to dislocation generation at an early stage in the process. This sample was prepared to demonstrate the potential of the presented technique for larger sample dimensions.

### 2.1. Composition of X-ray spectra

The measurements were conducted according to the following procedure utilizing the above-mentioned µ-XRF system. Point spectra were measured as a function of the rotation about the sample surface to find a suitable orientation that leads to a spectrum containing several intense diffraction maxima. This was done at ambient pressure using a custom stepper motor driven sample stage [Guguschev *et al.* (2020 ▸), Fig. 6] with a step size of 10° and a range of 360°.

**Figure 3:**
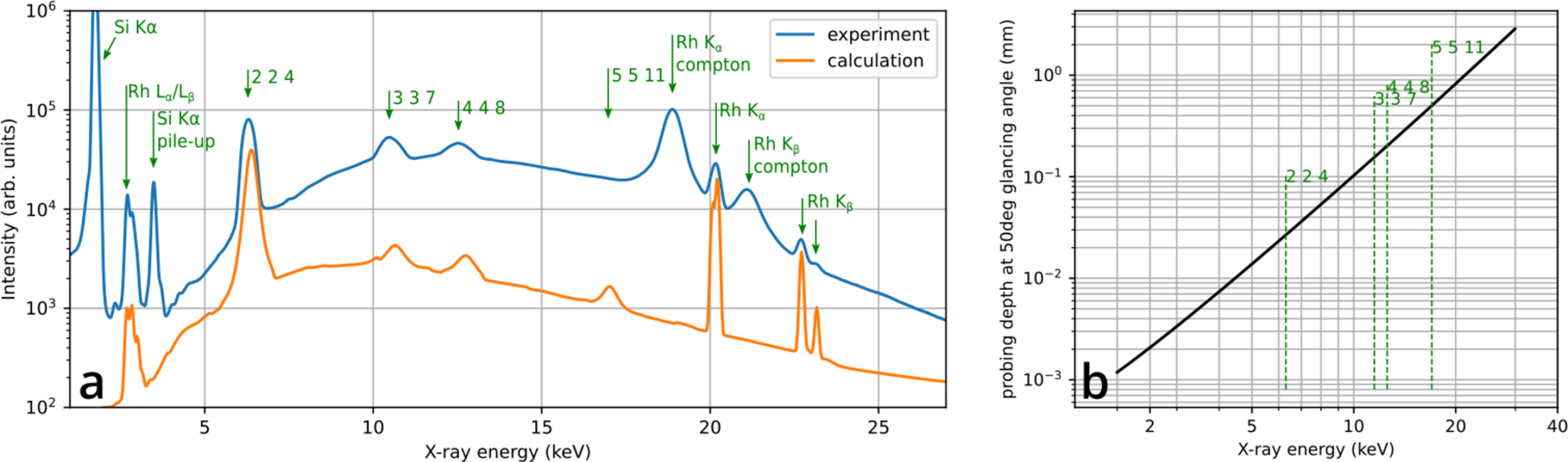
(*a*) A comparison between the measured and calculated spectra. (*b*) The probing depth in silicon based on the geometry (see Fig. 1) plotted as a function of energy.

In general, the spectra contain maxima due to characteristic X-ray fluorescence, Compton scattering and X-ray diffraction. For silicon, the characteristic emission is found at a rather low energy (around 1.8 keV and its pile-up at 3.6 keV) and therefore does not produce any features in the hard X-ray parts of the spectra [see Fig. 3 ▸(*a*)]. Diffraction maxima can occur for Bragg reflections that have lattice planes perpendicular to the bisector of the angle formed by the X-ray source, sample and detector. Therefore, in principle, only higher orders of the same fundamental reflection may be observed, which all have parallel lattice planes. However, due to the significant divergence of the beam (compare Fig. 2 ▸) and the large solid angle of the detector (0.075 sr), reflections from nearly parallel net planes can contribute. The diffraction maxima can be easily identified, as they change intensity when the crystal orientation is changed. The spectra in Fig. 3 ▸ also feature strong maxima at Rh emission and Compton peaks, which are due to the X-ray source being a Rh target and the Compton counterparts being shifted to lower energies because of inelastic scattering from the sample.

The simplified calculation in Fig. 3 ▸(*a*) is based on the excitation spectrum *I*_0_(*E*) of the X-ray source and the calculated positions of diffraction maxima. Considering the low angular resolution of the measurement, dynamic diffraction effects are neglected. The positions and intensities of the Bragg peaks were calculated using the Python package *xrayutilities* (Kriegner *et al.*, 2013 ▸), where the decay in intensity when deviating from the Bragg condition was taken into account according to Weckert & Hümmer (1997 ▸). The spectra are thus obtained by summing over all Bragg reflections *hkl* weighted by their structure amplitudes *F_hkl_* as follows:

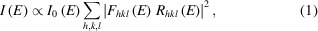

with the resonance term

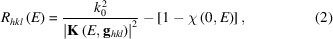

and **K** = 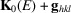 = 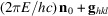 . Here, **n**_0_ is the direction of the incoming beam and **K**_0_ and **K** are the wavevectors of the incoming and diffracted beams, respectively (see Fig. 1 ▸). The summation over the reciprocal-lattice vectors **g** *_hkl_* needs to be limited such that the resulting diffracted beam **K** deviates by less than 17° from the direction of the detector, as defined by the solid angle of the latter, which amounts to 0.075 sr. An angular spread of **K**_0_ according to the incoming beam divergence (see Fig. 2 ▸) also needs to be considered. The term [1 − χ], where χ(0) is the zeroth Fourier component of the susceptibility, takes account of refraction and attenuation of the beam. Extinction and the finite intrinsic Darwin width of the reflections are neglected for simplicity. Since the scattering angle 2θ is fixed for all energies, we neglect the Lorentz and polarization factors.

The crystal orientation, which is needed for the calculation, was taken from a previously measured miscut of sample Si-1 of approximately 1° and refined regarding the rotation about the surface normal in order to reproduce the measurement. Not aiming at a quantitative fitting of the measurement, the calculation neglects factors such as the absorption by X-ray windows, the detector efficiency, the energy dependence of the efficiency of the optics and multiple scattering effects. This may be the reason why the predicted 5 5 11 reflection was not observed in the present experimental configuration. The probing depth for a specific reflection [Fig. 3 ▸(*b*)] equates to the projected attenuation length λ of the fluorescent X-rays that leave the sample under the glancing angle α (Fig. 1 ▸) and is thus calculated via λsin(α). Since λ depends on the X-ray energy, so does the penetration depth, as shown in Fig. 3 ▸(*b*). The 224 reflection is connected to a probing depth of approximately 30 µm, which roughly corresponds to the lateral resolution of the measurement. Structure deformations (bending or strain due to defects or compositional changes) within the probing depth are expected to change the intensities or positions of the diffraction maxima.

## 3. Results

### 3.1. Measurement of polished sections of Si crystals

**Figure 4:**
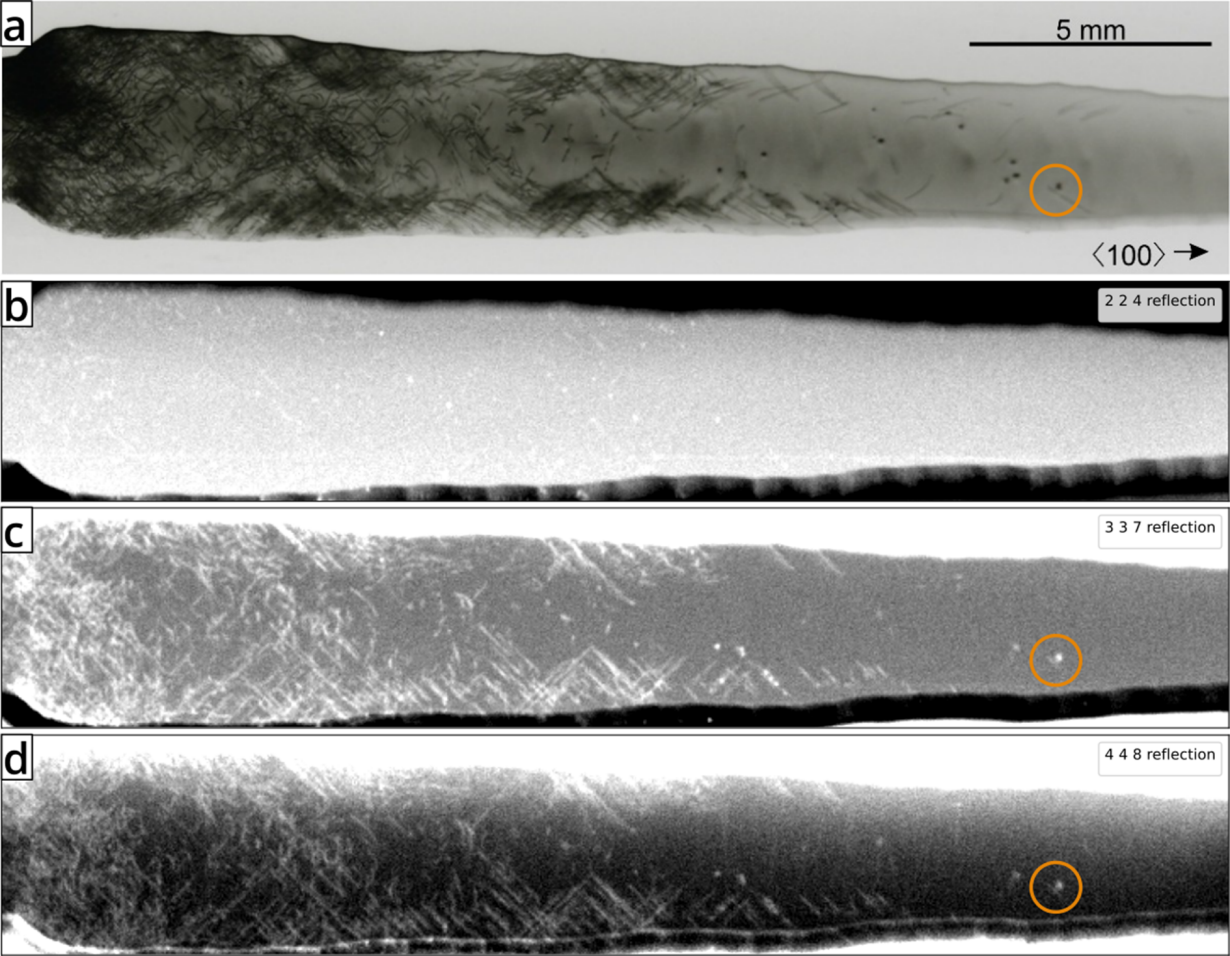
Defects in a (001) Si cross-section sample (sample Si-1). (*a*) A Lang topography image (004 reflection, Mo *K*α_1_ radiation). Adapted with permission from Dadzis *et al.* (2020 ▸) under a Creative Commons Attribution 4.0 International License, https://creativecommons.org/licenses/by/4.0/. (*b*)–(*d*) Maps of the diffracted intensity measured in the µ-XRF system for the selected maxima of the 224, 337 and 448 reflections, respectively. Increased brightness indicates an increase in the integrated intensity for the energy range corresponding to the indicated reflections.

The samples were investigated according to the measurement conditions shown in Table 1 ▸. Figs. 4 ▸(*b*)–4 ▸(*d*) show the integrated intensities of the 224, 337 and 448 Bragg reflections [Fig. 3 ▸(*a*)] as a function of sample position in comparison to a Lang topograph of the 004 reflection [Fig. 4 ▸(*a*)]. It is obvious that most of the line defects (dislocations) that are observed in the Lang topograph are also reproduced in the EDLM maps [Figs. 4 ▸(*b*)–4 ▸(*d*)]. Areas of the crystal with high, moderate and low dislocation density can be clearly distinguished from dislocation-free parts by both techniques. The locally deformed structure around defects leads to a broader acceptance angle for diffraction in comparison to the intrinsic Darwin width of the reflection. In conjunction with the broad bandwidth of the X-ray source, this results in an increase in the integrated diffracted intensity.

Some differences in the images in Figs. 4 ▸(*a*)–4 ▸(*d*) may be explained by the choice of reflections and the probing depth of the method. The Burgers vectors **b** of dislocations in the diamond structure of Si are of the kind {110}. The visibility criterion **b**·**g** = 0 [see *e.g.* Lider (2021 ▸)] states that the diffraction contrast due to dislocations is minimal if the reciprocal-lattice vector **g** *_hkl_*, corresponding to the observed Bragg reflection, is perpendicular to the Burgers vectors **b**. Therefore, neither the 004 reflection, used in the Lang topograph, nor the *hhl* reflections, mapped by EDLM here, will show all dislocations of the {110} family of Burgers vectors. Hence, a one-to-one correspondence of dislocations seen in EDLM and Lang topography is not expected. Nevertheless, many such cases can be identified where even a single dis­location is observed in both EDLM and Lang topography (*e.g.* highlighted by the orange circles in Fig. 4 ▸).

The dislocations mapped by EDLM exhibit a shorter length than those in the Lang topography data, which is due to the lower probing depth [see Fig. 3 ▸(*b*)]. As expected, the apparent dislocation lengths increase with higher orders of the reflection, although the difference between the 337 and 448 reflections is marginal. The low probing depth of the 224 reflection makes many dislocations appear as point-like features. Cases where the features appear elongated could be explained by a dislocation segment with the line direction being parallel to the surface, most likely along the predominant 〈110〉 directions.

**Figure 5:**
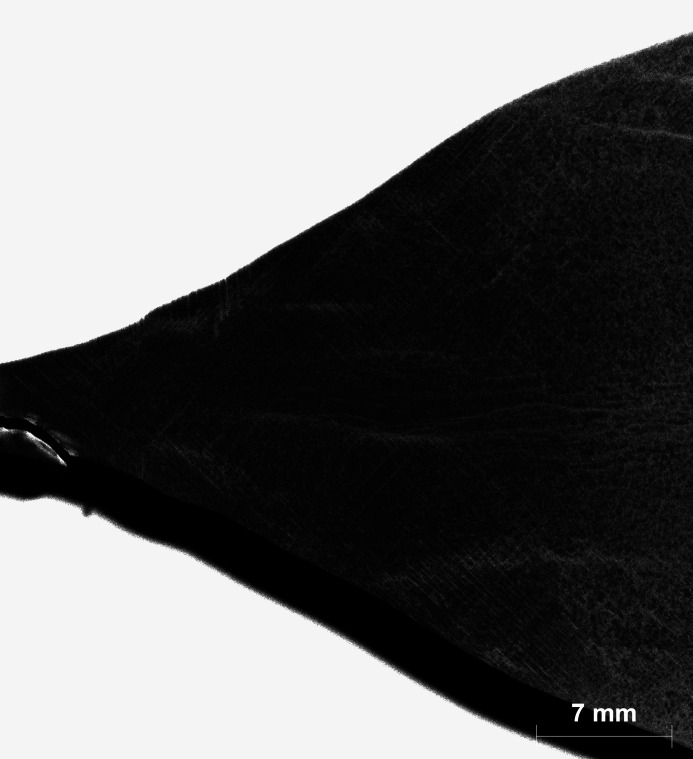
A superimposed greyscale-coded 2D intensity plot of the peak flanks and maxima of the 337 and 448 reflections of sample Si-2.

The presented technique also has potential for larger samples at reduced measurement times per pixel and larger distances between the individual measurements, as can be seen by the mapping result of sample Si-2 in Fig. 5 ▸. Here, the superimposed intensities of peak flanks and peak maxima of the 337 and 448 reflections are plotted. The left part of the sample is situated close to the seed and this region has the lowest dislocation density for this crystal. Nevertheless, it is important to avoid these initial dislocations, since multiplication of dislocations has occurred (visible in the central part) followed by the formation of subgrains and the appearance of cellular structures.

### 3.2. Measurement of a crystal boule with the AMS

**Figure 6:**
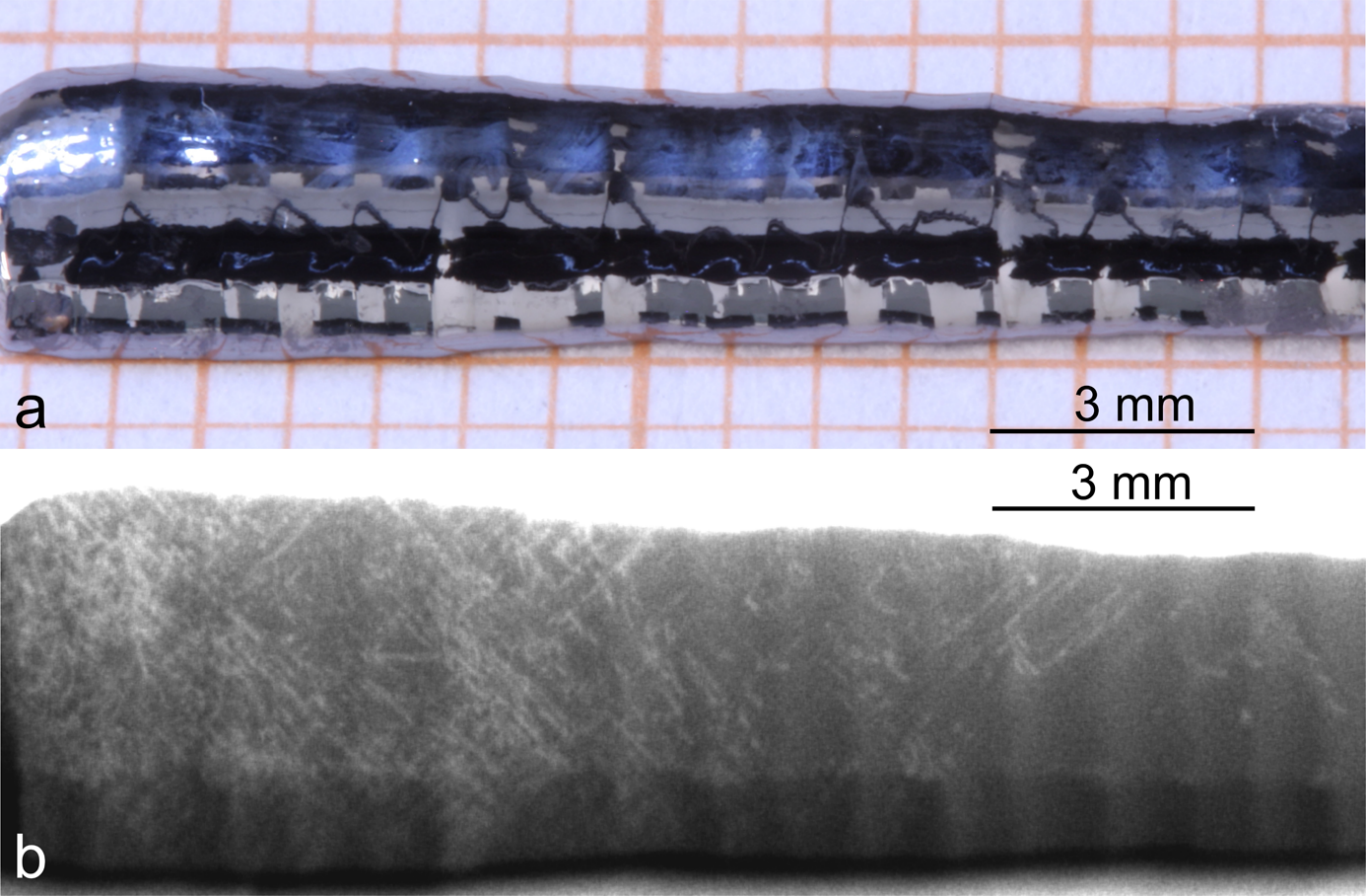
(*a*) A photograph of the investigated region of sample Si-3 with a similar viewing direction to the X-ray beam. (*b*) A superimposed greyscale-coded 2D intensity plot of the peak flanks and peak maxima of the 337 and 448 reflections of the sample shown in panel (*a*) with a slight increase in contrast (Rh source with AMS).

Using the AMS in combination with the presented energy-dispersive µ-XRF setup, it was possible to visualize dis­locations in an etched sample (Si-3) of the small-diameter bulk part of the boule [Fig. 6 ▸(*b*)]. The sample shape [Fig. 6 ▸(*a*)] was nearly as-cut, with a radius of curvature between 1.54 and 2.23 mm. The distribution of dislocations in the outer curved part could be imaged despite a very pronounced surface profile. The resulting distribution is comparable to that obtained for the neighbouring sample shown in Fig. 4 ▸.

## 4. Conclusion and outlook

It has been demonstrated that a benchtop µ-XRF laboratory device can be used to image individual line defects in a semiconductor material with a relatively high spatial resolution of ∼25 µm. The probed volume, which imposes an upper limit to the resolvable dislocation density, depends on this lateral resolution and the probing depth, which, in turn, is a function of the peak energy [Figs. 2 ▸ and 3 ▸(*b*)].

The results obtained for the Dash neck of an Si crystal have verified that most of the details visible in Lang topography can be visualized by EDLM. Given the widespread availability of µ-XRF devices, the described method­ology can facilitate the non-destructive high-throughput identification of defects in high-quality single crystals. The AMS improves the visualization of dislocations even for unpolished highly curved samples by decreasing the X-ray beam divergence. This enables the investigation of samples without the need for surface preparation. While non-planar surfaces distort the wavefield in standard XRT techniques due to an inhomogeneous phase shift that is a consequence of surface height variations, this is not a critical issue for the presented energy-dispersive technique, since it is realized by scanning with a focused convergent beam. Another advantage of the EDLM approach might be that the penetration depth into the material, which depends on the X-ray energy used, can be readily tuned by the choice of Bragg reflection or by an adjustment of the sample orientation, while the measurement geometry is fixed. This means that this technique enables defect imaging of crystals with various shapes and thicknesses, which is normally difficult in conventional XRT.

Further improvements of the technique would be possible according to simulations as shown in Fig. 3 ▸(*a*), allowing the determination of the sample orientation giving the strongest Bragg reflections and contrast due to the dislocations based on their Burgers vectors. The measurement time and the AMS setting have not yet been optimized in the demonstrated examples, which suggests that a higher throughput should be feasible. Possible limitations of the technique include the necessity of separating the information from diffraction and fluorescence. For some materials, the Bragg peaks can overlap with the characteristic X-rays for certain sample orientations.

In principle, this technique could be applied universally to all single-crystalline materials, but the penetration depth will be significantly reduced for denser materials such as GaAs or CdTe. While increasing the penetration capability by using higher energies is possible, this approach will soon reach its limits, since the intensity of the required high-order Bragg reflections will be lower and the fluorescence signal will increase due to the presence of heavier elements. Using additional detectors will allow the simultaneous recording of a large set of non-collinear Bragg reflections and hence enable the probing of dislocations with other Burgers vectors according to the visibility criterion as mentioned above.

## Figures and Tables

**Table 1:** Measurement conditions for high-resolution Bragg-peak mapping at a pressure of 20 mbar

Sample	Si-1 (polished)	Si-2 (polished)	Si-3 (etched)
Surface orientation	(100)	(100)	(100)
Mapping area (mm)	25.4 × 4.57	37.71 × 39.14	15.6 × 5.09
AMS in use	No	No	Yes (1000 µm aperture)
Data points (No. of pixels)	3629 × 653	2746 × 3008	2229 × 727
Distance between spots (µm)	7	13	7
Spot size (µm)	∼25	∼25	∼25
Cycles	1	1	1
Dwell time per point (ms)	70	14	40
Linear speed (µm s^−1^)	100	929	175
Total measuring time (h:min)	47:00	33:43	18:40

## References

[bb1] Becker, M., Regula, G., Reinhart, G., Boller, E., Valade, J.-P., Rack, A., Tafforeau, P. & Mangelinck-Noël, N. (2019). *J. Appl. Cryst.* **52**, 1312–1320.

[bb2] Buegler, M., Tagle, R., Reinhardt, F., Menzies, A. & Hill, T. (2021). *Microsc. Microanal.* **27**, 2208–2209.

[bb3] Dadzis, K., Menzel, R., Juda, U., Irmscher, K., Kranert, C., Müller, M., Ehrl, M., Weingärtner, R., Reimann, C., Abrosimov, N. & Riemann, H. (2020). *J. Electron. Mater.* **49**, 5120–5132.

[bb4] Danilewsky, A. N. (2020). *Cryst. Res. Technol.* **55**, 2000012.

[bb5] Dresselhaus-Marais, L. E., Winther, G., Howard, M., Gonzalez, A., Breckling, S. R., Yildirim, C., Cook, P. K., Kutsal, M., Simons, H., Detlefs, C., Eggert, J. H. & Poulsen, H. F. (2021). *Sci. Adv.* **7**, eabe8311. 10.1126/sciadv.abe8311 PMC8279502 34261647

[bb6] Fujie, F., Peng, H., Ailihumaer, T., Raghothamachar, B., Dudley, M., Harada, S., Tagawa, M. & Ujihara, T. (2021). *Acta Mater.* **208**, 116746.

[bb7] Guguschev, C., Klimm, D., Brützam, M., Gesing, T., Gogolin, M., Paik, H., Dittmar, A., Fratello, V. & Schlom, D. (2019). *J. Cryst. Growth*, **528**, 125263.

[bb8] Guguschev, C., Klimm, D., Brützam, M., Gesing, T., Gogolin, M., Paik, H., Markurt, T., Kok, D., Kwasniewski, A., Jendritzki, U. & Schlom, D. G. (2020). *J. Cryst. Growth*, **536**, 125526.

[bb9] Guguschev, C., Richter, C., Brützam, M., Dadzis, K., Hirschle, C., Gesing, T. M., Schulze, M., Kwasniewski, A., Schreuer, J. & Schlom, D. G. (2022). *Cryst. Growth Des.* **22**, 2557–2568.

[bb10] Guguschev, C., Tagle, R., Juda, U. & Kwasniewski, A. (2015). *J. Appl. Cryst.* **48**, 1883–1888.

[bb11] Hänschke, D., Danilewsky, A., Helfen, L., Hamann, E. & Baumbach, T. (2017). *Phys. Rev. Lett.* **119**, 215504. 10.1103/PhysRevLett.119.215504 29219418

[bb12] Hartmann, C., Kabukcuoglu, M. P., Richter, C., Klump, A., Schulz, D., Juda, U., Bickermann, M., Hänschke, D., Schröder, T. & Straubinger, T. (2023). *Appl. Phys. Expr.* **16**, 075502.

[bb13] Kirste, L., Grabianska, K., Kucharski, R., Sochacki, T., Lucznik, B. & Bockowski, M. (2021). *Materials*, **14**, 5472. 10.3390/ma14195472 PMC8509523 34639870

[bb14] Kriegner, D., Wintersberger, E. & Stangl, J. (2013). *J. Appl. Cryst.* **46**, 1162–1170. 10.1107/S0021889813017214 PMC3769072 24046508

[bb15] Lang, A. R. (1959). *Acta Cryst.* **12**, 249–250.

[bb16] Lider, V. V. (2021). *Phys. Solid State*, **63**, 189–214.

[bb17] Purushottam Raj Purohit, R. R. P., Tardif, S., Castelnau, O., Eymery, J., Guinebretière, R., Robach, O., Ors, T. & Micha, J.-S. (2022). *J. Appl. Cryst.* **55**, 737–750. 10.1107/S1600576722004198 PMC9348891 35974740

[bb18] Robach, O., Micha, J.-S., Ulrich, O. & Gergaud, P. (2011). *J. Appl. Cryst.* **44**, 688–696.

[bb19] Shikata, S., Miyajima, K. & Akashi, N. (2021). *Diamond Relat. Mater.* **118**, 108502.

[bb20] Simpson, C. A., Kozuki, S., Lopez-Crespo, P., Mostafavi, M., Connolley, T. & Withers, P. J. (2019). *J. Mech. Phys. Solids*, **124**, 392–410.

[bb21] Stockmeier, L., Lehmann, L., Miller, A., Reimann, C. & Friedrich, J. (2017). *Cryst. Res. Technol.* **52**, 1600373.

[bb22] Straubinger, T., Hartmann, C., Kabukcuoglu, M. P., Albrecht, M., Bickermann, M., Klump, A., Bode, S., Hamann, E., Haaga, S., Hurst, M., Schröder, T., Hänschke, D. & Richter, C. (2023). *Cryst. Growth Des.* **23**, 1538–1546.

[bb23] Subramanian, A., Abrosimov, N., Gybin, A., Guguschev, C., Juda, U., Fiedler, A., Bärwolf, F., Costina, I., Kwasniewski, A., Dittmar, A. & Sumathi, R. R. (2023). *J. Electron. Mater.* **52**, 5178–5188.

[bb24] Suvorov, E. V. (2018). *J. Surf. Investig.* **12**, 835–852.

[bb25] Tran Caliste, T. N., Kirste, L. & Baruchel, J. (2023). *Microelectron. Eng.* **276**, 112012.

[bb26] Weckert, E. & Hümmer, K. (1997). *Acta Cryst.* A**53**, 108–143.

[bb27] Yao, Y., Tsusaka, Y., Hirano, K., Sasaki, K., Kuramata, A., Sugawara, Y. & Ishikawa, Y. (2023). *J. Appl. Phys.* **134**, 155104.

[bb28] Yildirim, C., Poulsen, H. F., Winther, G., Detlefs, C., Huang, P. H. & Dresselhaus-Marais, L. E. (2023). *Sci. Rep.* **13**, 3834. 10.1038/s41598-023-30767-w PMC9992398 36882517

[bb29] Yoneyama, A., Ishiji, K., Sakaki, A., Kobayashi, Y., Inaba, M., Fukuda, K., Konishi, K., Shima, A. & Takamatsu, D. (2023). *Sci. Rep.* **13**, 12381. 10.1038/s41598-023-39347-4 PMC10390543 37524763

